# The Selectivity of Immunoassays and of Biomimetic Binding Assays with Imprinted Polymers

**DOI:** 10.3390/ijms221910552

**Published:** 2021-09-29

**Authors:** Gergely Becskereki, George Horvai, Blanka Tóth

**Affiliations:** Department of Inorganic and Analytical Chemistry, Budapest University of Technology and Economics, H-1111 Budapest, Hungary; becske3@gmail.com (G.B.); toth.blanka@vbk.bme.hu (B.T.)

**Keywords:** binding assay, antibody, interference, cross-reactivity, IC50, competitive, homogeneous, heterogeneous, tracer, binding curve

## Abstract

Molecularly imprinted polymers have been shown to be useful in competitive biomimetic binding assays. Recent developments in materials science have further enhanced the capabilities of imprinted polymers. Binding assays, biological and biomimetic alike, owe their usefulness to their selectivity. The selectivity of competitive binding assays has been characterized with the cross-reactivity, which is usually expressed as the ratio of the measured IC50 concentration values of the interferent and the analyte, respectively. Yet this cross-reactivity is only a rough estimate of analytical selectivity. The relationship between cross-reactivity and analytical selectivity has apparently not been thoroughly investigated. The present work shows that this relationship depends on the underlying model of the competitive binding assay. For the simple but widely adopted model, where analyte and interferent compete for a single kind of binding site, we provide a simple formula for analytical selectivity. For reasons of an apparent mathematical problem, this formula had not been found before. We also show the relationship between analytical selectivity and cross-reactivity. Selectivity is also shown to depend on the directly measured quantity, e.g., the bound fraction of the tracer. For those cases where the one-site competitive model is not valid, a practical procedure is adopted to estimate the analytical selectivity. This procedure is then used to analyze the example of the competitive two-site binding model, which has been the main model for describing molecularly imprinted polymer behavior. The results of this work provide a solid foundation for assay development.

## 1. Introduction

Molecularly imprinted polymers (MIP) [1,2,3,4,5,6,7,8,9,10,11,12,13,14,15] may be considered as bioinspired materials for a variety of reasons. They are macromolecules, often constitute a part of a nanocomposite system, and they can interact selectively with either small molecules, or macromolecules, or whole microorganisms, just like their biological counterparts. One of the main recent trends in MIP research has been the combination of a wide range of nanomaterials with MIPs. Furthermore, MIPs have been themselves prepared on the nanoscale. Nanocomposites have been shown to have improved features compared to more conventional MIPs. While there has been impressive progress in the design of ever newer and more complex MIP materials, the understanding of one of the main features of MIPs, their selectivity has not kept pace with the preparative developments [16,17].

The physical and chemical structure of the MIP binding sites has remained largely unknown. For this reason, one knows very little about the exact origin of MIP selectivity. Empirical methods for characterizing MIP selectivity vary according to the intended application. A fairly general method is the comparison of the binding isotherm of the analyte with that of a likely interferent [7]. In this method, no mixtures of the two compounds are studied, while in real life applications the two substances would be present together in the samples.

An important application of MIPs is in binding assays [18,19,20,21,22,23,24]. MIPs have been indeed regarded as artificial antibodies. It has also been claimed that the selectivity of certain MIPs is similar to that of the antibody it might replace. This has been proven by measuring the cross-reactivity of the MIP. This occurred by comparing the respective IC50 values (concentrations causing 50% inhibition of tracer binding) of the antibody and the MIP, respectively.

Despite the widespread use of the IC50 based cross-reactivity, both in bioanalysis and with MIPs, it appears difficult to find any proof that it really does what it promises, i.e., that it quantitatively characterizes the analytical selectivity of binding assays. Yet selectivity is very important in bioanalysis, i.e., in the analysis of small compounds or large biomolecules in biological samples [25].

In the present work we derive an exact formula for the selectivity of those binding assays which follow the widely used competitive binding equilibrium model on a single kind of binding site. It is shown that the selectivity depends on the value of the measured quantity, e.g., on the bound fraction, *B*, of the tracer. In contrast to this, the ratio of the IC50 value of the interferent to that of the analyte, is a single number, and thus it cannot characterize the selectivity, which depends on the bound fraction. Nevertheless, as shown in this paper, in a well-designed, single-site binding assay, the IC50 ratio is a reasonable approximation for the selectivity. Furthermore, it is shown, that “relative potency”, as defined by Ekins [26] is equal to the analytical selectivity if the single site model is valid.

The situation changes, however, if the single binding site model is not applicable. This is a common situation with MIPs. For this case, a general characterization method of binding assay selectivity has been adopted here. It is shown then, by example of a two-site binding model, that selectivity may change non-monotonously with *B*, and may go through a maximum. In the two-site model, neither the IC50 ratio nor the relative potency describes selectivity exactly.

The present work provides a solid basis for the assessment of the selectivity of biological and artificial binding assays.

## 2. Results

### 2.1. The One-Site Model of the Competitive Homogeneous Binding Assay

A homogeneous (in the sense of homogeneous phase reactions in chemistry) competitive binding assay relies on the use of a tracer, T. The tracer is typically, but not exclusively, the radiotracer version A* of the analyte, A. It will be assumed in the first part of this paper that there is only one type of binding site in the system, and this site will be denoted here by S. To discuss selectivity, an interfering compound X, which can react with the binding site S, will also be considered.

The simultaneous reactions between these components are as follows:

(1) T+S=TS

(2) A+S=AS

(3) X+S=XS

The equilibrium constants of these reactions are (with square brackets denoting equilibrium concentrations of the bracketed species):

(4) $$K_{T}=\frac{\left[TS\right]}{\left[T\right]*\left[S\right]}$$

(5) $$K_{A}=\frac{\left[AS\right]}{\left[A\right]*\left[S\right]}$$

(6) $$K_{X}=\frac{\left[XS\right]}{\left[X\right]*\left[S\right]}$$

The total concentrations of S, T, A, and X, respectively, will be denoted with *c_S_*, *c_T_*, *c_A_*, and *c_X_*. The mass balances for each component are:

(7) $$c_{S}=\left[S\right]+\left[TS\right]+\left[AS\right]+\left[XS\right]$$

(8) $$c_{T}=\left[T\right]+\left[TS\right]$$

(9) $$c_{A}=\left[A\right]+\left[AS\right]$$

(10) $$c_{X}=\left[X\right]+\left[XS\right]$$

In a typical assay, the total concentrations of S, *c_S_*, and of T, *c_T_*, are the same in all calibration and assay mixtures. The measured quantity may be either one of the following:

- the concentration of the bound tracer, [*TS*],
- the concentration of the free tracer, [*T*],
- the bound fraction of the tracer, *B* = [*TS*]/*c_T_*
- the ratio of the bound tracer concentration to the free tracer concentration, [*TS*]/[*T*].

Since the total concentration of the tracer is fixed and (usually also) known, either one of these measurable quantities allows the calculation of all others.

The one-site competitive binding model presented above has been known [27,28] to be only a simplified model of competitive homogeneous immunoassays. In spite of this, it has been used quite generally as a useful first approximation. Questions about the validity of the model will be addressed in a later section of the present paper. The model will also be extended to heterogeneous phase competitive assays, such as the biomimetic binding assays with molecularly imprinted polymers, or assays with antibodies fixed on a solid surface.

### 2.2. Quantitative Expressions of Assay Selectivity

**Figure 1:**
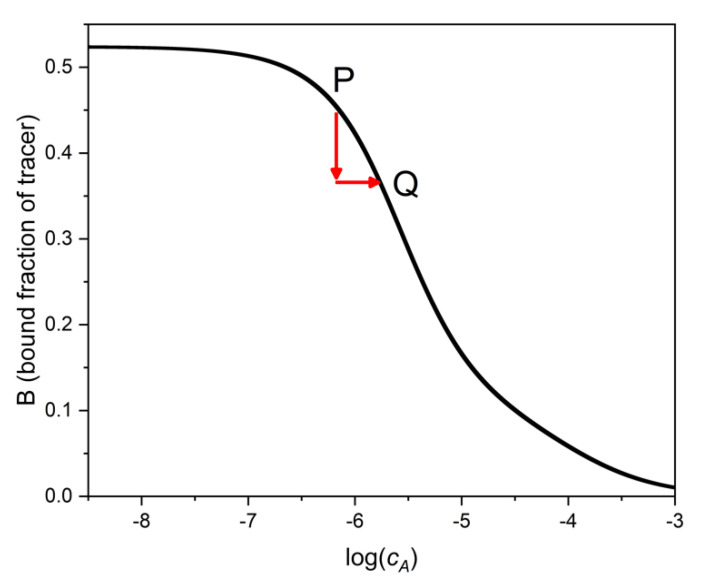
Binding curve of the analyte A. If interferent is present in a sample, the bound fraction of the tracer is lower than in the absence of interferent (vertical arrow). Since the concentration of A is estimated from this lower *B* value (as point Q), the estimated analyte concentration is higher than the true value. The error (more precisely the log ratio of estimated to true value) is shown by the horizontal arrow. This figure is general, i.e., not restricted to a particular binding model.

The main idea of the binding assay is that the bound (or the free) fraction of the tracer depends on the concentration of the analyte A. This is so because the tracer and the analyte compete for the fixed quantity of binding sites. In the absence of interferents, the dependence of tracer binding (e.g., of *B* = [*TS*]/*c_T_*) on the analyte’s total concentration, *c_A_*, or its logarithm, is the calibration function (also called “binding curve”) of the assay (Figure 1). In the presence of an interferent, X, at concentration *c_X_*, the calibration curve is no more valid. In practice, we are either unaware of the presence of the interferent, or we have no means to remove its effect. Thus, we read the concentration of A at the *B* value measured in the presence of both A and X, but from the calibration curve, which had been established in the absence of X. The determined apparent analyte concentration will be obviously in error. 

Figure 1 shows, that by taking a sample containing only A (point P in the figure), and then adding X to the sample, the binding value, *B*, decreases. If one reads the apparent concentration of A at the new *B* level from the binding curve of pure A (i.e., at point Q), the estimated sample concentration, *c_A_*(Q), will be higher than the true concentration *c_A_*(P).

The analyst’s goal is to estimate the error *c_A_*(Q) − *c_A_*(P), and to keep it within some predetermined tolerance limits. If this error in the analyte determination is small, even if the interferent concentration, *c_X_*, is high, then the assay is selective. It seems therefore meaningful to characterize the assay selectivity by the quantity

(11) $$SEL\left(c_{A},c_{X}\right)=\frac{c_{X}}{c_{A}^{\prime }-c_{A}}$$

where we denote *c_A_*(Q) with *c_A_*′ and *c_A_*(P) with *c_A_* for simplicity. Note, however, that this ratio, and thus the selectivity, is generally not a constant, because it depends on *c_A_* and on *c_X_*.

Bioanalysts had sought a single number to characterize the selectivity of a binding assay. They had proposed, and indeed have been using for decades, the concentration ratio IC50_X_/IC50_A_ of the binding assay, as the measure of its selectivity. Later, we shall explain this selectivity concept in detail. For now, it is only noted that even in the simplest assay model, the selectivity SEL, as defined above, is not a constant and it is not equal to IC50_X_/IC50_A_.

### 2.3. Derivation of the Selectivity of the One-Site Competitive Binding Assay

In this section, we shall derive a mathematical expression of the selectivity, SEL, in terms of the equilibrium constants and the other fixed parameters of the one-site assay. 

As noted above, if one measures the bound fraction, *B*, of the tracer, one can calculate also [*TS*] and [*T*] from the mass balance of the tracer. These values, in turn, determine through the equilibrium condition of the tracer (Equation (4)) the concentration of the free binding sites, [*S*]. With [*S*] known, one can use the respective mass balance and equilibrium equations of A and X to express [*AS*] and [*XS*] as a function of [*S*], and of the respective total concentrations *c_A_* and *c_X_*. Inserting these values into the mass balance equation of the sites:

(12) $$c_{S}=\left[S\right]+\left[TS\right]+\frac{c_{A}}{1+\frac{1}{K_{A}*\left[S\right]}}+\frac{c_{X}}{1+\frac{1}{K_{X}*\left[S\right]}}$$

The relationship between [*S*] and the measured quantity, *B*, is:

(13) $$B=\frac{\left[TS\right]}{c_{T}}=\frac{\frac{c_{T}}{1+\frac{1}{K_{T}*\left[S\right]}}}{c_{T}}=\frac{1}{1+\frac{1}{K_{T}*\left[S\right]}}$$

and expressing [*S*] with *B*:

(14) $$\left[S\right]=\frac{B}{K_{T}\left(1-B\right)}$$

Substituting this result into Equation (12):

(15) $$c_{S}=\frac{B}{K_{T}*\left(1-B\right)}+c_{T}*B+\frac{c_{A}}{1+\frac{K_{T}*\left(1-B\right)}{K_{A}*B}}+\frac{c_{X}}{1+\frac{K_{T}*\left(1-B\right)}{K_{X}*B}}$$

This equation contains now only the measured quantity *B*, the known (or at least constant) quantities *c_S_* and *c_T_*, and finally the total (analytical) concentrations of the analyte, *c_A_*, and of the interferent, *c_X_*. If *c_X_* = 0, then the equation is the calibration curve (binding curve) of the assay in an implicit form, because it describes the relationship between *c_A_* and B. If *c_A_* = 0, then the equation is the interferent’s calibration curve. If neither *c_A_* nor *c_X_* is zero, the equation describes the relationship between the measured *B* and the two total (analytical) concentrations, *c_A_* and *c_X_*. In this case *B* is a bivariate function, and therefore its dependence on *c_A_* and *c_X_* together can be plotted either as a 3D surface, or as a series of contour lines in the (*c_A_*, *c_X_*) plane.

It would be convenient to obtain the *B*(*c_A_*, *c_X_*) function in explicit form, i.e., to express *B* as a function of *c_A_* and *c_X_*. However, this would lead to a very complicated trigonometric expression [29] because the equation is cubic in *B*.

This is the salient point, where earlier studies had stopped, and could not derive a simple expression for the selectivity of the assay. To move forward from here, we use our recent result [16,30], which tells that the selectivity of any analytical method can be defined by a unique constant if and only if the response variable of the method (in this case *B*) is an univariate function of a linear combination (i.e., additive linear function) of the analyte and interferent concentrations, respectively, with constant coefficients. Actually, the response function need not be an explicit function of the concentrations, it may also have the following implicit form, as adapted for the present case:

(16) $$f(B,\;\left(k_{A}*c_{A}+k_{X}*c_{X}\right)=0$$

where *f* is an arbitrary bivariate function of *B* and (k_A_ ∗ *c_A_* + k_B_ ∗ *c_B_*), and where k_A_ and k_X_ are nonnegative constants.

When Equation (16) is valid for the assay, then the selectivity of the assay may be calculated using the procedure described above in relation to Figure 1. One needs to equate the response *B* in the (*c_A_*, *c_X_*) mixture with the response of a pure A solution of concentration *c_A_*′:

(17) $$B\left(c_{A},\;c_{X}\right)=B\left(c_{A}\prime \right)$$

From this and from Equation (16):

(18) $$k_{A}*c_{A}^{\prime }=k_{A}*c_{A}+k_{X}*c_{X}$$

Hence,

(19) $$SEL=\frac{c_{X}}{c_{A}^{\prime }-c_{A}}=\frac{k_{A}}{k_{X}}$$

The bivariate binding function, Equation (15) would satisfy the general equation (16) if the denominators of *c_A_* and *c_X_* were constant values, not functions of the variable *B*. (Note that *c_S_*, *c_T_* and K_T_ are constants, not variables in the assay).

Now one can use a simple trick. Consider Equation (15) separately for each value of the measured *B*. For any fixed *B*, the denominators of *c_A_* and *c_X_* are constants, and the equation describes a contour line of the three dimensional *B*(*c_A_*, *c_X_*) function. As the equation shows, the contour lines are straight lines, which are parallel with the (*c_A_*, *c_X_*) plane. The selectivity of the assay is constant along these lines, and its value is the ratio of the respective multiplier of *c_A_* and *c_X_* in the equation:

(20) $$SEL\left(B\right)=\frac{1+\frac{K_{T}*\left(1-B\right)}{K_{X}*B}}{1+\frac{K_{T}*\left(1-B\right)}{K_{A}*B}}$$

This result shows, that the selectivity of the assay depends on the measured value *B*, and through it from [*S*], the concentration of free binding sites. This situation is completely analogous with the selectivity of ion-selective electrodes [31,32], which depends on the measured EMF value alone.

It is useful to calculate the selectivity of the binding assay at some particular values of *B*. If *B* is close to 1, i.e., when *c_A_* and *c_X_* are very low, and almost all tracer is bound, then SEL ≈ 1, as is easily seen from Equation (20). This means, that when almost no tracer has been displaced by the analyte and the interferent together, the assay cannot differentiate the analyte from the interferent. If *B* is close to zero, i.e., when almost all tracer is displaced by high analyte and/or interferent concentrations, SEL ≈ K_A_/K_X_. Thus, at high analyte concentration, the selectivity is high since typically K_A_ >> K_X_. Practical competitive assays often start from *B* ≈ 0.5. At this value of *B*, and assuming K_T_ ≈ K_A_ and K_A_ >> K_X_ (which is the case with many radio-immunoassays) SEL ≈ 0.5 ∗ K_A_/K_X_.

**Figure 2:**
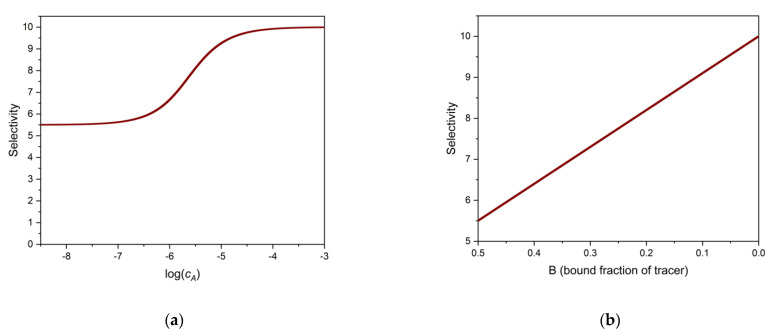
Selectivity of a one-site binding assay as a function of analyte log concentration (log *c_A_*) (**a**), and as a function of binding, *B* (**b**). Parameters used: *c_S_* = 1 × 10^−6^ M, K_A_ = 1 × 10^6^ M^−1^, K_X_ = 1 × 10^5^ M^−1^. K_T_ = K_A_ is assumed.

These examples show that the selectivity of the binding assay depends very much on the value of *B*. Figure 2a. shows the selectivity as the function of the analyte’s log concentration, and Figure 2b shows the selectivity, calculated from Equation (20), as a function of *B*. The parameters of the calculations may be found in the figure legend. Note the strict linear relationship between SEL and *B*. 

In view of Equation (16), if the simple one-site competitive model is valid, the analytical error, *c_A_*′ − *c_A_*, which is due to a small addition of interferent, may be simply expressed as:

(21) $$c_{A}^{\prime }-c_{A}=\frac{c_{X}}{SEL\left(B\right)}$$

At any measured *B* value, the *SEL*(*B*) value can be calculated from Equation (20). The equilibrium constants, needed for this calculation, can be obtained from the separate calibration (binding) curves of the analyte and the interferent, respectively.

## 3. Discussion

### 3.1. Expressing the Selectivity with IC50 Values and with Relative Potency

**Figure 3:**
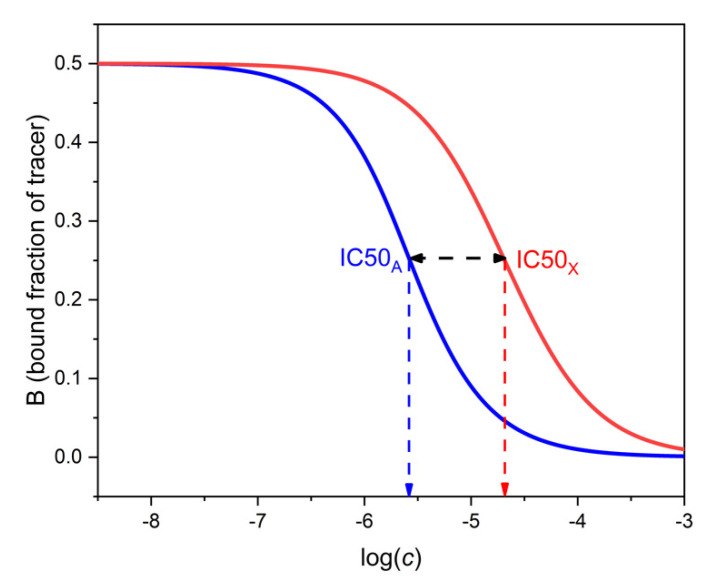
Determination of cross-reactivity from the binding curves of the analyte A (left, blue) and the interferent X (right, red). The respective log IC50 values can be read at 0.5 ∗ *B*_0_ = 0.25 as shown. The log of the cross-reactivity is shown by the horizontal black double-headed arrow. Parameters as in Figure 2.

The selectivity of binding assays has been traditionally expressed by the cross-reactivity. This is the ratio of the respective IC50 values of the analyte and the interferent. IC50 is the concentration of the respective compound which decreases *B* to 50% of *B*_0_, where *B*_0_ is the value of *B* measured at *c_A_* = *c_X_* = 0, i.e., in the absence of both analyte and interferent. Typical competitive immunoassays are designed to have *B*_0_ ≈ 0.5. In this case, IC50_A_ is the concentration of the analyte which pertains to *B* ≈ 0.25 on the analyte’s binding curve. IC50_X_ is the concentration of the interferent which pertains to *B* ≈ 0.25 on the interferent’s binding curve. Figure 3 shows calibration lines and IC50 values calculated with realistic parameters.

The cross-reactivity, i.e., the concentration ratio IC50_X_/IC50_A_, can be calculated by expressing *c_A_* from Equation (15) for *B* = 0.25 and *c_X_* = 0, and comparing the value with *c_X_* obtained from the same equation for *B* = 0.25 and *c_A_* = 0. By doing this calculation, one obtains (assuming again K_T_ ≈ K_A_ and K_A_ >> K_X_) that the cross-reactivity is approximately equal to 0.775 K_A_/K_X_. This is three quarters of the maximal selectivity.

As noted earlier, the analytical selectivity, SEL, depends on *B*. If we calculate its value at *B* = 0.25 from Equation (20), it is found to be 0.775 K_A_/K_X_, i.e., equal with the cross-reactivity. This is not a mere coincidence, as will be shown in the next section.

### 3.2. What Does the IC50 Ratio Tell about Assay Selectivity?

The ratio of the IC50 values of an interferent and the analyte, IC50_X_/IC50_A_, has been typically used to characterize the assay selectivity. For example, if the ratio was 20, then it was concluded that the interference level was 5%. In other words, it was assumed that the analytical bias, *c_A_*′ − *c_A_*, caused by X was 0.05 ∗ *c_X_*, independently from the value of the actual concentrations or of *B*.

A problem with this assumption had been recognized, but not widely known, in the immunoassay literature (but was probably not noticed at all in the MIP literature). Regarding immunoassays, Ekins [33] wrote: “Regrettably, the observation that the potency of a cross-reacting substance relative to the analyte is not a constant has been obscured by the common practice of reporting the relative amounts of cross-reactant and analyte which reduce the zero dose response (e.g., *B*_0_) by 50%, this quantity sometimes being referred to as the ‘coefficient of cross reactivity’ (CR_50%_). However, for several reasons, the value of this coefficient may substantially misrepresent the biasing effect of the cross reactant in the system.”

Ekins had recognized that the “relative potency”, i.e., the ratio of *c_X_* to *c_A_*, as read at a common B value from the respective calibration lines (binding curves) of A and X, respectively, depends on the *B* value where these readings are made. He also recognized that the *c_X_*/*c_A_* ratio is related to the analytical bias. However, he and others had only assumed and not proved that the bias (*c_A_*′ − *c_A_* in our notation) at any binding value *B* was proportional to the relative potency at this *B*. Yet this can be easily proved using our Equation (15). The binding curve for A in the absence of X is simply Equation (15) with *c_X_* = 0. Similarly, the binding curve of X in the absence of A is Equation (15) with *c_A_* = 0. Expressing *c_A_* from the first of these equations and *c_X_* from the second, while using the same *B*, and forming the ratio *c_X_*/*c_A_* with these values, one obtains *SEL*(*B*), as given in Equation (20). This proves that the relative potency at any *B* is equal to *SEL*(*B*). Note, however, that this result has been derived here with the one-site model. For other models it may not be valid. It will indeed be shown below that for a two-site binding model it is not valid.

### 3.3. Biomimetic Binding Assays with MIPs

Molecularly imprinted polymers (MIP) have been used as artificial antibodies since the early 1990-s [19,20]. Mosbach’s group presented several successful competitive binding assays with MIPs [8,18], and these were followed by others. The MIPs, at least in the version used by these pioneers, were solid sorbents. Their binding behavior resembled that of immobilized antibodies. Yet it has been debated for decades [7] if a MIP has one, two, or many different types of binding sites, of which only one would participate in the immunoassays, or there is a continuous spectrum of binding sites.

The selectivity of the MIP binding assays has been characterized by IC50 ratios. This had been done apparently merely by analogy with immunoassays. One may ask therefore, if this practice is justified, and if so, if it has the same problems, which had been noted above for homogeneous assays. The main difference from the homogeneous assays discussed above is, that the binding sites, S, and the bound forms of T, A, and X, i.e., ST, SA, and SX, are all in (or on the surface of) the solid phase.

If one assumes that in the MIP binding assay only one kind of binding site participates, and the analyte has to compete for a limited amount of such sites with the tracer, and with an interferent, then one can obtain similar equations to those found above for homogeneous phase binding assays. If we denote solid phase/surface concentrations by overbars, the equilibrium equations will be:

(22) $$K_{T}=\frac{\left[\bar{TS}\right]}{\left[T\right]*\left[\bar{S}\right]}$$

(23) $$K_{A}=\frac{\left[\bar{AS}\right]}{\left[A\right]*\left[\bar{S}\right]}$$

(24) $$K_{X}=\frac{\left[\bar{XS}\right]}{\left[X\right]*\left[\bar{S}\right]}$$

The mass balance equations will be as follows:

(25) $${\bar{q}}_{S}=\left[\bar{S}\right]+\left[\bar{TS}\right]+\left[\bar{AS}\right]+\left[\bar{XS}\right]$$

(26) $$V*c_{T}=V*\left[T\right]+m*\left[\bar{TS}\right]$$

(27) $$V*c_{A}=V*\left[A\right]+m*\left[\bar{AS}\right]$$

(28) $$V*c_{X}=V*\left[X\right]+m*\left[\bar{XS}\right]$$

where *q_s_* is the total site concentration in the solid phase related to unit mass of the solid, *m* is the mass of the solid phase and *V* is the volume of the liquid phase. Note that *c_T_*, *c_A_* and *c_X_* denote total concentrations of the respective species in solution before equilibrating the solution(s) with the solid.

From these equations one can derive the same relationship, Equation (15), between the bound fraction of the tracer and the total sample concentrations *c_A_* and *c_X_*, as before. One may go through the whole derivation again, or one may simply note that by division of the new mass balance equations by *V*, and applying the following replacements, one gets automatically the same result as above in Equation (15):

(29) $$\frac{m}{V}*\left[\bar{TS}\right]=\left[TS\right]$$

(30) $$\frac{m}{V}*\left[\bar{AS}\right]=\left[AS\right]$$

(31) $$\frac{m}{V}*\left[\bar{XS}\right]=\left[XS\right]$$

(32) $$\frac{m}{V}*{\bar{q}}_{S}=c_{S}$$

(33) $$\frac{m}{V}*\left[\bar{S}\right]=\left[S\right]$$

With these notations, *B* and *SEL* have the same formulas as above in Equations (15) and (20). This proves that the conclusions made above for the selectivity of one-site homogeneous binding assays are also valid for the heterogeneous phase one-site binding assays including the biomimetic MIP binding assays.

### 3.4. The Limitations of the Competitive One-Site Model

In the above derivations it has been assumed that the binding of the tracer, of the analyte and of an interferent occurs competitively on a single kind of binding site. Real systems may differ from this simple model in many ways. Some of these are as follows:

- There may be more than one kind of binding site in the system.
- The binding sites, even of the same kind, may not be independent from each other, i.e., binding at one site may influence the binding at another site.
- The stoichiometry of binding may be different from the simple 1:1 stoichiometry assumed above.
- There may be more than one interferent present, and their effects may not be additive.
- The interferent may interfere with the measurement by a different mechanism from competitive binding, e.g., it may react with the analyte.

In the next section, the first of these problems will be discussed, and it will be assumed that there are two different kinds of binding site present. This case is particularly important for MIPs, which are often described as having two kinds of binding site: one strong and selective but at low concentration, and another which is weaker, less selective (or not selective at all), but more abundant. 

### 3.5. The Competitive Model with Two Sites

As noted above, one limitation of the model used in the first part of this paper is, that only one kind of binding site is considered. Now, we shall show that for competitive binding at two different binding sites, the results may be surprisingly different.

The competitive binding of analyte A and interferent X will be simulated for the case when there are two types of binding site, S1 and S2, of different selectivities and concentrations in the system. To simplify the discussion, it will be assumed here that the tracer is chemically identical with the analyte, and the tracer concentration is negligible. Thus, *B* will be equal to the fraction of analyte bound to both sites together.

A relatively simple binding equation, like Equation (15), could not be derived for the two-site case. One can use, however, a simple method to calculate the binding curves even in this case.

In the Appendix A of this paper it is shown how one can calculate *B* for any mixture of A and X. As special cases, the equations of the separate binding curves of A and X are also derived. This allows to read from the curves the IC50 values, the crossreactivity, as well as the relative potency as a function of B. The analytical selectivity, SEL, is also obtained for any mixture of A and X. 

**Figure 4:**
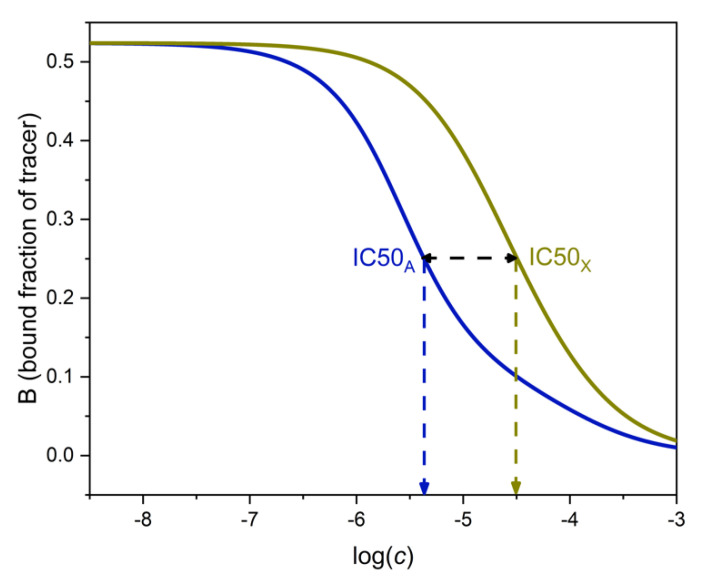
Determination of the cross-reactivity of a two-site, heterogeneous phase binding assay from the binding curves of the analyte A (left, blue) and the interferent X (right, green). The parameters of the curves are shown in Table 1.

Using the results of the Appendix A, the binding curves of A and X have been calculated for a hypothetical (but realistic) MIP, and are shown in Figure 4. The parameters used in the calculation are shown in Table 1. The phase ratio, i.e., the ratio of solution volume to sorbent mass has been selected as 1000.

As seen from Table 1, site 1 has higher binding constant (*K_A_*_1_) for A than for X (*K_X_*_1_), but on site 2 the binding constants are equal. This means that site 1 is selective for the analyte, but site 2 is not. The concentration of site 1 is ten times lower than the concentration of site 2. Such relationship is fairly typical for MIPs.

Figure 4 shows the separate binding curves of A and X, respectively. The IC50 values are shown on the corresponding curves.

**Figure 5:**
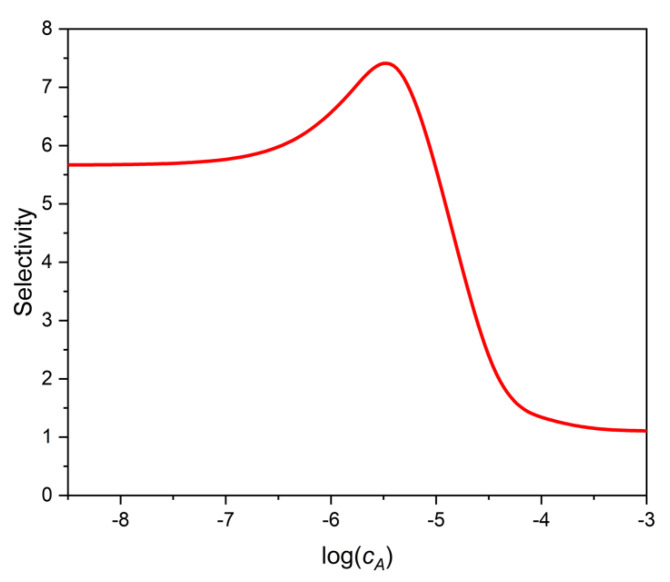
Selectivity of a two-site, heterogeneous phase binding assay as a function of analyte log concentration (log *c_A_*). The parameters used are shown in Table 1. Calculations were made with low enough interferent concentrations for the selectivity to be constant at any given *c_A_*.

Figure 5 shows the selectivity of the two-site binding assay as a function of log *c_A_*, the logarithmic analytical concentration of the analyte A. (The selectivity, SEL, has been calculated directly with the definition of selectivity, Equation (11), for small interference levels, where SEL did not depend significantly on *c_X_*). One can see that as *c_A_* increases, or as B decreases, the selectivity increases at first, similarly to the one-site case discussed earlier. However, between log *c_A_* = −6 and −5, the selectivity begins to decrease, and finally flattens. The reason for this behavior is, that the selectivity of the weaker sites is lower (*K_A_*_2_/*K_X_*_2_ = 1), than that of the stronger sites (*K_A_*_1_/*K_X_*_1_ = 10). The strong sites dominate adsorption at low concentrations, and their selectivity improves as *c_A_* increases. The weak, and in this case not selective, sites become more important at high concentrations, and the overall selectivity shows the curious behavior presented in Figure 5.

The ratio IC50_X_/IC50_A_ as read from Figure 4 is 7.3. This value is the same as the highest selectivity value (7.3) in Figure 5. Therefore, the selectivity estimate based on IC50 values would be too optimistic as one moves away from the maximum point of Figure 5 in either direction.

One may also compare the *B*-dependent SEL values with the *B*-dependent relative potency values for the two-site competitive model. In the example of Figure 4 and Figure 5, the two values begin to deviate substantially from each other when *B*/*B*_0_ is less than about 0.2 (data not shown). When *B*/*B*_0_ is about 0.1, SEL is only about half of the relative potency. This is a marked difference compared to the single-site case, where SEL was equal to the relative potency.

The results in this section have been derived for heterogeneous phase binding assays, but they are also valid for homogeneous phase binding assays, due to the use of essentially the same equilibrium and mass balance equations.

### 3.6. A Practical Approach to Estimate the Selectivity of Binding Assays

In view of the many possible deviations from the simple model, there is probably no general procedure possible for estimating selectivity. To circumvent at least some of the problems, one may employ the following procedure, which is directly based on the definition of selectivity in Equation (11). After measuring the calibration curve of the assay for the analyte in interferent-free solutions, one can spike the analyte solutions at a few calibration points with the interferent, possibly at two spiking levels in each point. After addition of the interferent, one can determine for the spiked solution an apparent analyte concentration, *c_A_*′, from the pure analyte’s binding curve, as had been shown on Figure 1. The difference between this apparent concentration and the true analyte concentration of the spiked sample is the error of the analyte measurement, caused by the addition of the interferent, X, as presented in Figure 1. The error due to X is:

(34) $$error\;of\;c_{A}=c_{A}^{\prime }-c_{A}$$

For small errors, one may assume (or experimentally check with a different spike) that the error is proportional to *c_X_*, so that the selectivity is meaningfully characterized by

(35) $$SEL=\frac{c_{X}}{c_{A}^{\prime }-c_{A}}$$

From these measurements, one can derive a plot similar to that shown in Figure 5. This is a useful information for optimizing the assay. The American Association of Pharmaceutical Scientists proposed a similar procedure for immunoassays [34], but no comparison between this method and the IC50 method seems to have been made. As far as we know, in the field of biomimetic MIP binding assays the problems with IC50 values as selectivity estimates have not yet been recognized, and the here suggested procedure has not been tested.

The procedure described here does not increase the number of necessary measurements to obtain information about binding assay selectivity, because it makes the measurement of the binding curve of the interferent X superfluous. Moreover, it is free from any assumptions concerning the binding assay model.

The usefulness of the here proposed method is also underlined by the result of the previous section, where it had been shown, that even for the relatively simple two-site model, neither the cross-reactivity nor the relative potency can correctly predict the analytical selectivity.

## 4. Conclusions

Competitive binding assays are considered to be among the important achievements with MIPs. The selectivity of these binding assays has been characterized in the literature with the ratio of the measured IC50 values of the interferent and the analyte, respectively. This practice has been borrowed from the literature of immunoassays. Yet the ratio of IC50 values is only a rough estimate of selectivity. This difficulty had been recognized by some authors with respect to immunoassays, but much less with the biomimetic MIP binding assays. 

For reasons of an apparent mathematical complexity, no general formula had been found earlier for the selectivity of competitive binding assays, be they biological or MIP based. The present work has shown how this difficulty can be overcome. As a result, a simple formula has been provided to calculate the selectivity of the one-site competitive binding assays, as a function of a directly measured quantity, like the bound fraction of the tracer. The calculated, concentration (or binding ratio) dependent selectivity of the assay has been compared to IC50 ratios and to relative potencies. It has been shown that IC50 ratios are only rough estimates of selectivity, while relative potency is accurate, but only if the one-site model is valid.

For those cases where the simple competitive model with a single kind of binding site is not valid, a practical procedure has been proposed to estimate the selectivity. The usefulness of this procedure has been demonstrated for binding assays where two different binding sites are present. This example showed also that the assay selectivity may vary non-monotonously in the useful concentration range of the assay. Finally, the deviation of the “relative potency” of a binding assay from its analytical selectivity, has been demonstrated for the two-site case.

The results of the paper have been shown to apply to immunological and MIP-based biomimetic assays as well. They are equally valid for assays in homogeneous solutions and for assays where the binding sites are in a solid phase or on the surface of a solid material. Therefore, the present work provides a solid basis for the development of a broad range of assays. It shows also that some conventional selectivity measures, which are used in established analytical methodologies, e.g., competitive binding assays, may have a complex relationship with the analytical error caused by the interferents. Analysis of selectivity measures, as used elsewhere in (bio)analytical chemistry, may be worth a similar study to the present one.

## Figures and Tables

**Table 1:** Parameters of a two-site, heterogeneous phase binding assay. The unit of *q_site_* is mol/kg, and the unit of K is M^−1^. K_T_ = K_A_ is assumed.

Site Number	*q_site_*	K_A_	K_X_
1	1.00 × 10^−3^	1.00 × 10^6^	1.00 × 10^5^
2	1.00 × 10^−2^	1.00 × 10^4^	1.00 × 10^4^

## Data Availability

Not applicable.

## Appendix A

Let us consider first only one of the two sites, say S1. For this site, one can write the same equilibrium and mass balance equations as have been written above for the one-site model. Only the notation of the quantities needs to be modified. For simplicity, only that case will be considered, where the tracer is chemically identical with the analyte, and the tracer concentration is negligible compared to the analyte concentration. Thus,

(A1) $$A+S_{1}=AS_{1}$$

(A2) $$A+X_{1}=AX_{1}$$

The corresponding equilibrium constants will be denoted by *K_A_*_1_ and *K_X_*_1_, respectively.

The total concentration of S1, A and X will be denoted with *c_S_*_1_, *c_A_* and *c_X_*, respectively. The mass balance for S1 is:

(A3) $$c_{S1}=\left[S_{1}\right]+\left[AS_{1}\right]+\left[XS_{1}\right]=\left[S_{1}\right]\left(1+K_{A1}\left[A\right]+K_{X1}\left[X\right]\right)$$

Expressing [*S*_1_] from this equation and introducing it into the equilibrium expressions of A and X, respectively, one obtains expressions for [*AS*_1_] and [*XS*_1_]. Here, only [*AS*_1_] is shown:

(A4) $$\left[AS_{1}\right]=\frac{c_{S1}K_{A1}\left[A\right]}{1+K_{A1}\left[A\right]+K_{X1}\left[X\right]}=D_{A1}\left[A\right]$$

where the notation *D_A_*_1_ has been introduced. This is a kind of distribution coefficient for the distribution of A between the bound form *AS*_1_ and the free form A, respectively. Note that *D_A_*_1_ can be calculated from [*A*], [*X*] and some constants.

Similar equations are obtained for [*XS*_1_], [*AS*_2_] and [*XS*_2_]. The mass balance for A is:

(A5) $$c_{A}=\left[A\right]+\left[AS_{1}\right]+\left[AS_{2}\right]=\left[A\right]\left(1+D_{A1}+D_{A2}\right)$$

The total adsorbed concentration of A is [*AS*_1_] + [*AS*_2_]. Thus, the bound fraction of A, and accordingly that of the tracer, is:

(A6) $$B=\frac{\left[AS_{1}\right]+\left[AS_{2}\right]}{c_{A}}=\frac{\left[A\right]\left(D_{A1}+D_{A2}\right)}{\left[A\right]\left(1+D_{A1}+D_{A2}\right)}=\frac{D_{A1}+D_{A2}}{1+D_{A1}+D_{A2}}$$

Since all *D*-s can be calculated if [*A*] and [*X*] are given, *B* can also be calculated from these two variables. The total concentration of A, *c_A_*, can also be calculated from these two variables, and *c_X_* similarly. Therefore, by taking an arbitrary ([*A*], [*X*]) pair, one can obtain *B*, *c_A_* and *c_X_*, i.e., *B* can be obtained as a function of the total concentrations *c_A_* and *c_X_*. The two binding curves can be obtained by setting [*X*] = 0 or [*A*] = 0 in the expressions for the relevant *D*-s.

For the calculation of *SEL* in a mixture of A and X, one needs to get *c_A_*′ for the above obtained *B* value of the mixture, but in the absence of X. Since from *B* one can calculate *D_A_*_1_ + *D_A_*_2_, and in the absence of X the value of *D_A_*_1_ + *D_A_*_2_ depends only on [*A*], one can obtain [*A*] from *B*, actually by solving a quadratic equation. From this [*A*], one then obtains *c_A_*′ as

(A7) $$c_{A}^{\prime }=\left[A\right]\left(1+D_{A1}+D_{A2}\right)$$

The selectivity is calculated finally as:

(A8) $$SEL=\frac{c_{X}}{c_{A}^{\prime }-c_{A}}$$

For any given *c_A_*, SEL may depend on *c_X_*. By calculating SEL for some analytically relevant values of *c_X_*, one can get a good picture of the expected level of interference.

It is worth noting, that the total bound concentration of A is:

(A9) $$\left[AS_{1}\right]+\left[AS_{2}\right]=\frac{c_{S1}K_{A1}\left[A\right]}{1+K_{A1}\left[A\right]+K_{X1}\left[X\right]}+\frac{c_{S2}K_{A2}\left[A\right]}{1+K_{A2}\left[A\right]+K_{X2}\left[X\right]}$$

This equation is formally identical with the so-called bi-Langmuir equation [35], which is used when A is adsorbed on a solid which has two independent binding sites. This observation shows that our results may be used for two-site binding both in homogeneous and heterogeneous phase, e.g., for solid state MIPs. To demonstrate this application, the numerical examples in the paper relate to such solid phase binding on two different binding sites.
